# Leukocyte DNA methylation in Alzheimer´s disease associated genes: replication of findings from neuronal cells

**DOI:** 10.1080/15592294.2022.2158285

**Published:** 2022-12-26

**Authors:** Ida K Karlsson, Alexander Ploner, Yunzhang Wang, Margaret Gatz, Nancy L Pedersen, Sara Hägg

**Affiliations:** a Department of Medical Epidemiology and Biostatistics, Karolinska Institutet, Stockholm, Sweden; b Center for Economic and Social Research, University of Southern California, Los Angeles, CA, USA; c Department of Psychology, University of Southern California, Los Angeles, CA, USA

**Keywords:** Dementia, Alzheimer’s disease, DNA methylation, twins

## Abstract

Differences in gene-wide DNA methylation of the Alzheimer’s disease (AD)-associated genes *BIN1, HLA-DRB5, SORL1, SLC24A4*, and *ABCA7* are reported to be associated with AD in post-mortem brain samples. We investigated whether the same associations could be found in leukocytes collected pre-mortem. Using cohort data of 544 Swedish twins (204 dementia diagnoses), we replicated the findings in *HLA-DRB5* and *SLC24A4* at *P* < 0.05. However, co-twin control analyses indicated that the associations were partly explained by familial confounding. Thus, DNA methylation differences in *HLA-DRB5* and *SLC24A4* are present in both neuronal cells and leukocytes, and not fully explained familial factors.

## Introduction

Epigenetic alterations in relation to Alzheimer’s disease (AD) have been studied extensively and evidence an important role of DNA methylation differences in both blood and brain samples in association with AD [1]. One previous study by Yu et al. applied a gene-wide approach to study changes in methylation across 28 genes associated with the disease in relation to AD using post-mortem brain samples [2]. They identified such changes in the *BIN1, HLA-DRB5, SORL1, SLC24A4*, and *ABCA7* loci. The association of disease with both allelic and DNA methylation variation of the genes highlight their importance in AD pathology. However, little to no work has examined DNA methylation of these genes in blood samples [1]. The aim of the current study was therefore to investigate whether the same associations are present in blood samples collected pre-mortem from dementia patients and controls. In addition, we aimed to examine if the associations are driven by genetic or other familial confounding, by studying the associations in dementia discordant twin pairs.

## Methods

The study population originated from sub-studies of ageing in the Swedish Twin Registry [3]: The Swedish Adoption/Twin Study of Ageing (SATSA) [4], Study of Dementia in Swedish Twins (HARMONY) [5], and TwinGene [3]. All participants provided informed consent and the studies were approved by the Regional Ethical Review Board in Stockholm. Dementia and AD information was available from clinical evaluations in SATSA and HARMONY and from nationwide registers for all participants (the National Patient Register, the Cause of Death Register, and the Prescribed Drug Register, updated through 2016) [6]. Dementia ascertainment is described in detail in the Supplementary material. To gain power, we used all dementia as the main outcome in this study, but also modelled AD separately.

Blood samples were collected as part of the studies. If samples from more than one time point were available, we selected the last available sample prior to disease onset for individuals with dementia, alternatively the first available after onset. To achieve a similar age at blood sample among controls, we used the last available sample.

Extracted DNA was bisulphite converted and analysed using the Infinium Human Methylation 450 K Bead Chip (*n* = 427), or the Infinium MethylationEPIC Bead Chip (*n* = 117), both from Illumina Inc., San Diego, CA, USA. The raw data were pre-processed using a multi-step quality control (QC) pipeline [7], including adjustment for batch effects and cell counts. *M*-values for all CpG sites which were located within each of the five genes of interest (±100 kb) and which passed QC on both methylation arrays were extracted (*n* = 368; Supplementary table S1).

Statistical analyses were performed in R 3.5.2, following the paper by Yu and colleagues [2]. For each retained CpG site, *M*-values were modelled as exposure for dementia or AD in logistic regression models, adjusting for age at blood sample, sex, and relatedness among twins (robust sandwich estimator). Data from the two methylation arrays were analysed separately, and combined using fixed-effect meta-analysis. For each gene, we then combined the *P*-values for all CpG sites within each gene into a test statistic X for gene-wide significance via Fisher’s method:

$$X=-2*\left(\sum_{k=0}^{n}log\left(p_{i}\right)\right)$$

where p_i_ denotes the *P*-value for the *i*th CpG. A one-sided *P*-value for the gene-level null hypothesis of no association of any CpG site with the outcome was calculated based on this test statistic using b = 1000 random permutations of the underlying data. *P*-values below α = 0.05 were considered statistically significant. The test procedure was repeated for the analysis of the discordant twin pairs, but based on conditional logistic regression models, and adjusting only for age at blood sample. Sensitivity analyses additionally adjusted from time between dementia diagnosis and blood sample for individuals diagnosed with dementia, and for time between mean age at dementia onset in the sample and blood sample for controls.

## Results

Among the 427 individuals analysed on the Illumina 450 K array, 140 individuals were diagnosed with dementia at a mean age of 80.7 y, 81 of whom were diagnosed with AD. Mean age at blood sample for the total sample was 78.1 y (77.2 for individuals with dementia, 80.0 for controls) and 60.7% were women. Among individuals with dementia diagnosis, 91 had blood samples collected prior to dementia diagnosis (mean 4.4 y) and 49 after dementia diagnosis (mean 4.4 y). The sample included 170 complete twin pairs (71 monozygotic; 99 dizygotic), out of which 42 pairs were discordant for dementia and 21 for AD.

Among the 117 individuals analysed on the Illumina EPIC array, 64 were diagnosed with dementia at a mean age of 78.4 y, 57 of whom were diagnosed with AD. Mean age at blood sample was 72.0 y (for both individuals with dementia and controls). All with dementia diagnosis had the blood sample collected prior to diagnosis (mean 6.3 y). The sample included 58 complete twin pairs (26 monozygotic; 32 dizygotic), out of which 53 pairs were discordant for dementia and 48 for AD.

**Figure 1.**
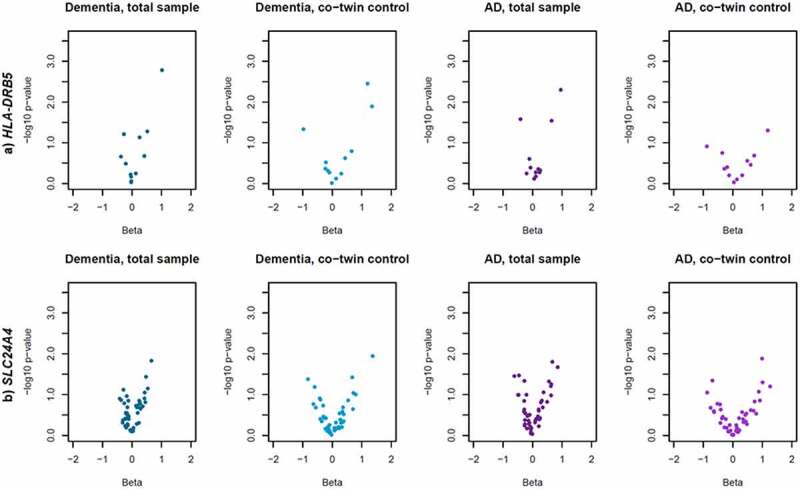
**Leukocyte DNA methylation in *HLA-DRB5* and *SLC24A4* in relation to dementia and Alzheimer’s disease**. Volcano plots of beta values and significance level from logistic regression models of leukocyte DNA methylation M-values at in a) *HLA-DRB5* and b) *SLC24A4* in relation to dementia and Alzheimer’s disease (AD). Each point represent one individual CpG site. Models using the total sample were adjusted for age at blood sample, sex, and relatedness among twins. Co-twin control models were adjusted for age at blood sample.

Differential leukocyte DNA methylation in association to dementia (at *P* < 0.05) was detected in *HLA-DRB5* and *SLC24A4* in the total sample. *HLA-DRB5* remained significant in co-twin control analyses, while the association between DNA methylation in *SLC24A4* and dementia was weakened (Table 1), indicating that familial confounding partly drive the association. It should be noted that the co-twin control association between DNA methylation in *HLA-DRB5* and dementia was largely driven by a single site, cg13022993, with a substantially stronger association in the co-twin control model (β = 1.20, *P* = 0.004) than in the full sample (β = 0.26, *P* = 0.074), while associations at other sites were weakened (Supplementary table S2b). Regression estimates and significance levels for all sites in *HLA-DRB5* and *SLC24A4* are visualized in Figure 1 (corresponding figures for the other genes are available in Supplementary Figure S1). Regression estimates, standard errors, and P-values for all individual CpG sites are presented in Supplementary table S2a-e. Results were consistent for AD, except that HLA-DRB5 was not significant in co-twin control analyses (Table 1). Sensitivity analyses adjusting for time between dementia diagnosis or mean age at dementia onset and blood sample (for individuals with or without a dementia diagnosis, respectively) were consistent with the main analyses, with P-values as follows: *BIN1*: 0.07; *HLA-DRB5*: 0.01; *SORL1*: 0.07; *SLC24A4*: 0.02; *ABCA7*: 0.24.

**Table 1.** Gene-level significance of DNA methylation in leukocytes in relation to dementia and Alzheimer’s disease.

		Dementia		Alzheimer’s disease	
	N CpG sites	Total sample	Co-twin control	Total sample	Co-twin control
*BIN1*	59	0.07	0.69	0.05	0.58
*HLA-DRB5*	12	**0.02**	**<0.01**	**0.04**	0.18
*SORL1*	47	0.06	0.75	0.10	0.96
*SLC24A4*	48	**0.01**	0.17	**0.01**	0.16
*ABCA7*	202	0.24	–	0.23	–

Number of CpG sites included and gene-level significance for each of the five genes. Logistic regression models were applied to test associations between CpG *M*-vaules and dementia or Alzheimer’s disease. Models using the total sample were adjusted for age at blood sample, sex, and relatedness among twins, and co-twin control models adjusted for age at blood sample. *P*-values for each CpG site across the genes were combined into a test statistic using the Fisher product method, and the statistical significance tested through random permutations. Bold numbers indicate significance at the α = 0.05 level. The co-twin control permutation test for ABCA7 did not converge.

## Discussion

Yu et al. [2] identified five AD related genes as differentially methylated in cortical cells from deceased AD patients compared to controls. We demonstrated that differences in DNA methylation of two of these genes, namely *HLA-DRB5* and *SLC24A4*, are also present in leukocytes pre-mortem. Differential methylation being present not only in the affected neuronal cells but also in the periphery, indicates that differences in the corresponding protein levels on the systemic level may be related to dementia and AD.

4The association between DNA methylation of *SLC24A4* and dementia or AD risk was weakened in co-twin control analyses, indicating that the association is partly, but not fully, driven by genetic or other familial confounding. The presence of familial confounding implies that DNA methylation of these genes is similar in twins discordant for dementia or AD, and thus explained by, e.g., genetic or early life confounders shared by the twins, rather than directly related to dementia or AD. In other words, there may be genetic or early life factors that explain both the DNA methylation of these genes and the development of dementia, rather than the DNA methylation directly attributing to dementia or AD. It is still possible that DNA methylation in these genes contribute to dementia pathological processes, if there is a shared environmental exposure that explains similarity in DNA methylation in the genes. Alternatively, there are other risk factors for which twins are discordant that explain why one twin has developed dementia while the other has not. The association with *HLA-DRB5* remained in twin pairs discordant for dementia, but not AD. This difference appear to be driven by a single CpG site, strongly associated with dementia in the co-twin control analyses but not with AD in either the full sample or co-twin control, while associations at other sites were weakened. The co-twin control results for dementia is thus likely driven by a single spurious finding, and taken together the results indicate the presence of familial confounding also in the association between *HLA-DRB5* and dementia or AD.

Our findings of no difference in DNA methylation of *SORL1* and *ABCA7* in relation to AD are in line with previous findings in blood [1]. However, there is some evidence that DNA methylation at specific CpG sites in *BIN1*, which was close to significance in the current study, differ in blood samples from AD patients compared to controls [8]. No previous studies have examined leukocyte DNA methylation of *HLA-DRB5* or *SLC24A4* in relation to AD. *HLA-DRB5* is involved in immunological response and *SLC24A4* in neuronal development, but little is known about how the genes affect AD risk [1].

Main strengths of this study are the well-characterized samples and the possibility to adjust for genetic and other familial influences through the twin design. DNA methylation is partly driven by genetic factors [9], and twin designs can make valuable contributions to the field as they elegantly account for effects driven by such genetic confounding. Although twins comprise the total sample as well as co-twin control sample, the results from the total sample analyses represent the population effect, i.e., approximates the estimate obtained if the sample included non-related individuals, while within twin pair analyses are fully adjusted for residual confounding shared by the twins (such as genetic or early environmental factors) [10]. As the estimates are drawn from the same sample, the results are directly comparable. A limitation of the method applied here is that it provides no effect estimate, only a gene-level *P*-value. As a result, we could not compare the effect estimate between the total sample and the co-twin control analyses, other than on the single-CpG level, and as there are rather few discordant twin pairs it is possible that power was simply too low to detect a significant signal within twin pairs. On the single-CpG level there was a larger spread of effect estimates in the co-twin control model compared to the total sample, indicating larger imprecision. It should also be noted that the method does not take correlation between CpG sites into account. However, as the permutation null distribution was derived from randomized phenotypes only, any such correlations are constant across permuted and real data, and the reported *P*-values therefore robust to underlying correlation structures. Moreover, CpG sites often cluster in CpG islands [1], acting together to regulate transcription, and DNA methylation levels even at correlated sites may act additively. Using only CpG sites that passed QC on both arrays limited the number of included sites, and fewer sites were included compared to the study by Yu et al. [2] (Supplementary Table S1). This may have led to lower power or spurious findings, especially for *HLA-DRB5* where only 12 sites were included in the current study, compared to 48 sites in the study by Yu et al. [2] Using dementia information from registers provides the possibility to follow individuals after end of study, but it should be noted that register-based diagnoses have excellent specificity, but rather low sensitivity [11]. The sample included blood samples collected both before and after disease diagnosis, and with a limited sample size we could not study gene-wide differences in DNA methylation before and after disease onset. Results were stable when adjusting for time between dementia onset and blood sample, but it should be noted that this adjustment cannot identify differences in associations before and after disease onset. We did not have access to post-mortem neuronal tissue and cannot confirm whether the originally reported associations are present in the current sample. We can therefore not rule out that the differences in findings are due to sample differences rather than tissue specificity.

In conclusion, *HLA-DRB5* and *SLC24A4* are not only differentially methylated in cortical cells from AD patients compared to controls, but also in leukocytes collected pre-mortem in relation to dementia. Although genetic or other familiar confounding appears to drive part of the associations, a difference in DNA methylation in association to dementia may remain even within twin pairs.

## Supplementary Material

Supplemental Material

## Data Availability

The SATSA data are available at National Archive of Computerized Data on Aging under accession number ICPSR 3843 (phenotypic data; https://www.icpsr.umich.edu/web/NACDA/studies/3843) and the EMBL‐EBI repository under accession number E‐MTAB‐7309 (DNA methylation data, 450K array; https://www.ebi.ac.uk/arrayexpress/experiments/E-MTAB-7309). Other data are held by the Swedish Twin Registry and can be applied for at https://ki.se/en/research/the-swedish-twin-registry. All codes used to generate analysis data and for conducting analyses are available upon request to the corresponding author.
