# Supplementary material for: Leukocyte DNA methylation in Alzheimer´s disease associated genes: replication of findings from neuronal cells
Source: Epigenetics. 2022 Dec 26;18(1):2158285. doi: 10.1080/15592294.2022.2158285 (PMC9980709; doi:10.1080/15592294.2022.2158285)
Supplement: Supplemental Material [file KEPI_A_2158285_SM7832.zip › supplement/Supplementary section_r1.docx]

**Dementia data in the Swedish Twin Registry**

**Clinical dementia ascertainment**

The SATSA, OTCTO-Twin, GENDER, and HARMONY studies included clinical dementia ascertainment (1). The in-person testing (IPT) phases in SATSA, OCTO-Twin, and GENDER included cognitive screening by using the Mini-Mental State Examination (2) together with additional cognitive tests. HARMONY used the TELE screening (3) administered during the telephone interview. Those who performed poorly on the TELE were complemented with informant interviews with the Blessed Dementia Rating Scale (BDRS)(4). All individuals suspected of dementia and their co-twins were referred to a clinical examination in HARMONY and the first three IPT phases in SATSA. The clinical examination included cognitive testing, physical and neurological work-ups, laboratory tests, reviews of medical records and informant interviews. In addition, for most cases computer tomography was added to aid in differential diagnosis, but led to little change in initial diagnosis (5). In GENDER, OCTO-Twin, and from the fifth IPT phase in SATSA, dementia diagnoses were based on review of medical records, the research nurse’s evaluation, and the extensive cognitive testing. For all sub-studies, final dementia diagnosis was set at multidisciplinary consensus conferences, according to DSM-III-R(6) or DSM-IV(7) criteria. Differential diagnosis of Alzheimer’s disease was set according to the NINCDS/ADRDA criteria (8).

**Dementia from national registers**

The STR is linked to several population-based registries through the 10-digit personal identification number assigned to all residents in Sweden. For the current study, dementia information was obtained from the Cause of Death Registry (CDR) and the National Patient Registry (NPR). Information about medication was retrieved from the Prescribed Drug Registry (PDR).

The NPR was initiated in 1964, and has since 1987 a nationwide coverage that includes information about 99% of all in-patient care at hospitals in Sweden(9). For each hospitalization, a recording of the primary diagnosis together with up to 20 additional diagnoses is done according to International Classification of Diseases (ICD) codes. The NPR has coverage of outpatient specialist care as well since 2001. Information about underlying and contributory causes of death for all Swedish residents has been included in the CDR since 1961, also reported according to ICD codes (10). Currently, data are available through the end of 2016 from NPR and CDR. Criteria for disease used both primary and additional diagnoses from the NPR, together with underlying and contributing causes of death from the CDR. The ICD codes used to retrieve dementia diagnoses are reported in Table S1. These codes were chosen to include chronic, progressive impairment of cognitive function.

The PDR has since 2005 information on all dispensed prescriptions classified according to the Anatomical Therapeutic Chemical (ATC) codes(11). The register is currently updated through the end of 2017, but to correspond to the information from the NPR and CDR for this study only information through 2016 was included. ATC codes for dementia medication are reported in Table S2.

**Table S1: ICD codes used to identify dementia**

| **ICD-7**  (used before 1969) | **ICD-8**  (used 1969-1986) | **ICD-9***  (used 1987-1996) | **ICD-10**  (used 1997 onwards) |
| --- | --- | --- | --- |
| **Alzheimer’s disease** | | | |
| **304** Senile psychosis | **290** Senile and presenile dementia | **290** Senile and presenile organic psychotic condition | **F00** Dementia in Alzheimer's disease |
| **305** Presenile psychosis |  | **294B/ 294.1** Dementia in conditions classified elsewhere | **G30** Alzheimer's disease |
|  |  | **331A/ 331.0** Alzheimer's disease |  |
| **Other dementia** | | | |
| **306** Psychosis with cerebral arteriosclerosis | **293.0** Cerebral arteriosclerosis | **331B/ 331.1** Pick's disease | **F01** Vascular dementia |
|  | **293.1** Other cerebrovascular disturbances | **331C/ 331.2** Senile degeneration of brain | **F02** Dementia in other diseases classified elsewhere |
|  |  | **331X/ 331.9** Cerebral degeneration, unspecified | **F03** Unspecified dementia |
|  |  |  | **F051** Delirium superimposed on dementia |
|  |  |  | **G311** Senile degeneration of brain, not elsewhere classified |
|  |  |  | **G318A** Other specified degenerative diseases of nervous system: Lewy body dementia |

* In the Swedish adaptation of ICD-9, the 4^th^ digit was replaced with a letter. While the NPR used the Swedish version, the international ICD version was used in CDR(10), and both versions are therefore reported here.

**Table S2: ATC-codes for identification of dementia medication**

| **N06DA** Anticholinesterases |
| --- |
| N06DA02 Donepezil |
| N06DA03 Rivastigmine |
| N06DA04 Galantamine |
| (N06DA01 Tacrine and N06DA05 Ipidacrine not prescribed in Sweden) |
| **N06DX** Other anti-dementia drugs |
| N06DX01 Memantine |
| (N06DX02 Ginkgo folium not prescribed in Sweden) |

**Figure S1: Leukocyte DNA methylation in *BIN1*, *SORL1*, and *ABCA7* in relation to dementia and Alzheimer’s disease**


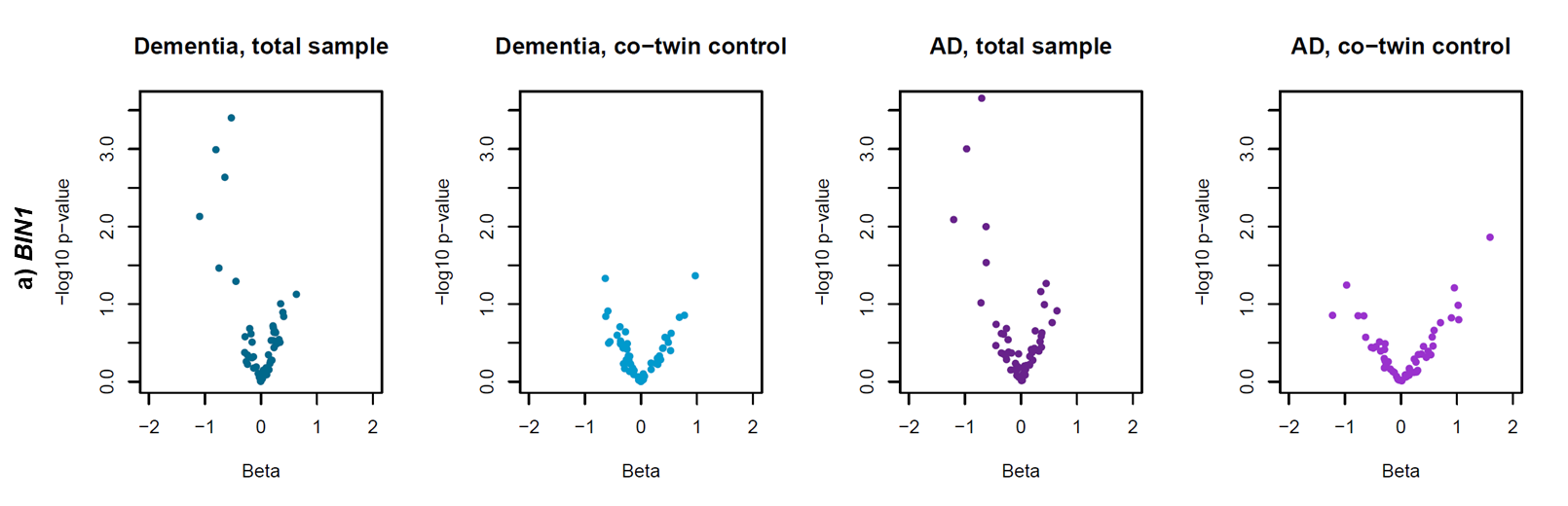


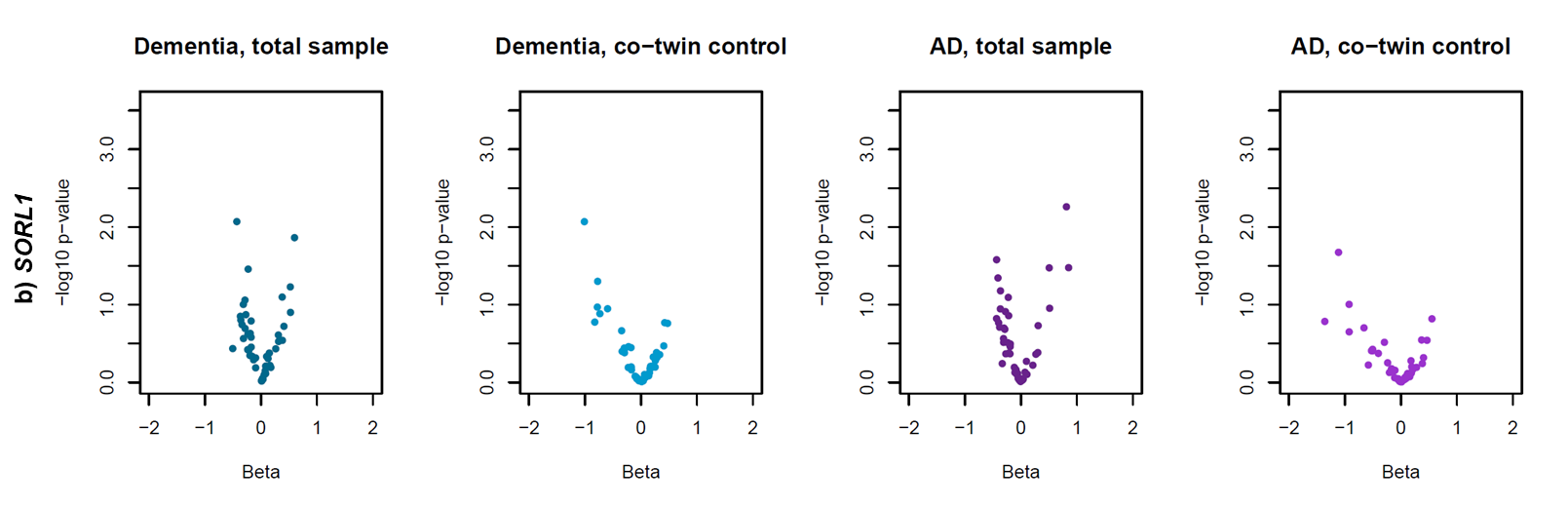


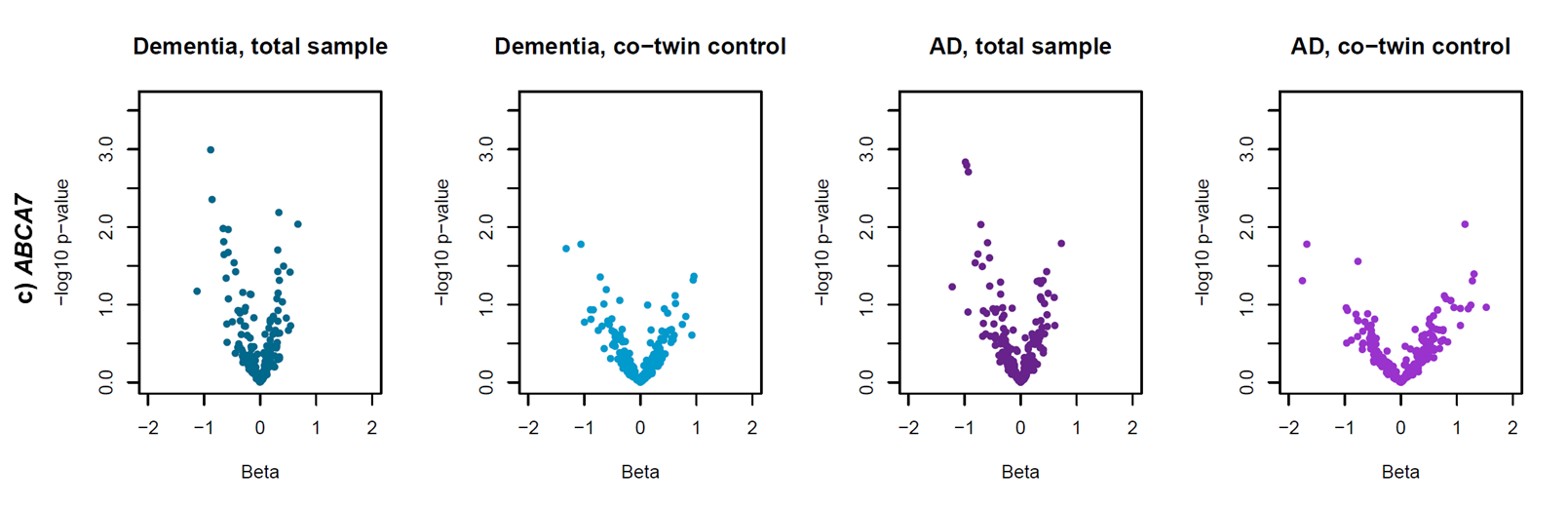


Volcano plots of beta values and significance level from logistic regression models of leukocyte DNA methylation M-values at in a) *BIN1*, b) *SORL1*, and c) *ABCA7* in relation to dementia and Alzheimer’s disease (AD). Each point represent one individual CpG site. Models using the total sample were adjusted for age at blood sample, sex, and relatedness among twins. Co-twin control models were adjusted for age at blood sample.

**References**

1. Gatz M, Fratiglioni L, Johansson B, Berg S, Mortimer JA, Reynolds CA, et al. Complete ascertainment of dementia in the Swedish Twin Registry: the HARMONY study. Neurobiology of Aging. 2005;26(4):439-47.

2. Folstein MF, Folstein SE, McHugh PR. "Mini-mental state". A practical method for grading the cognitive state of patients for the clinician. J Psychiatr Res. 1975;12(3):189-98.

3. Gatz M, Reynolds CA, John R, Johansson B, Mortimer JA, Pedersen NL. Telephone screening to identify potential dementia cases in a population-based sample of older adults. Int Psychogeriatr. 2002;14(3):273-89.

4. Blessed G, Tomlinson BE, Roth M. The association between quantitative measures of dementia and of senile change in the cerebral grey matter of elderly subjects. Br J Psychiatry. 1968;114(512):797-811.

5. Johansson B, Fratiglioni, L, Pedersen, NL, Gatz, M editor Does CT make a difference in diagnosis of dementia in epidemiological research? A comparison of diagnosis with and without CT. NorAge meeting; 2005; Norrköping, Sweden.

6. American Psychiatric Association. Diagnostic and statistical manual of mental disorders : DSM-III-R: American Psychiatric Association; 1987.

7. American Psychiatric Association. Diagnostic and statistical manual of mental disorders : DSM-IV: American Psychiatric Association; 1994.

8. McKhann G, Drachman D, Folstein M, Katzman R, Price D, Stadlan EM. Clinical diagnosis of Alzheimer's disease: report of the NINCDS-ADRDA Work Group under the auspices of Department of Health and Human Services Task Force on Alzheimer's Disease. Neurology. 1984;34(7):939-44.

9. Ludvigsson JF, Andersson E, Ekbom A, Feychting M, Kim JL, Reuterwall C, et al. External review and validation of the Swedish national inpatient register. BMC Public Health. 2011;11:450.

10. Brooke HL, Talback M, Hornblad J, Johansson LA, Ludvigsson JF, Druid H, et al. The Swedish cause of death register. European journal of epidemiology. 2017;32(9):765-73.

11. Wallerstedt SM, Wettermark B, Hoffmann M. The First Decade with the Swedish Prescribed Drug Register - A Systematic Review of the Output in the Scientific Literature. Basic Clin Pharmacol Toxicol. 2016;119(5):464-9.
